# The effects of midwives’ job satisfaction on burnout, intention to quit and turnover: a longitudinal study in Senegal

**DOI:** 10.1186/1478-4491-10-9

**Published:** 2012-04-30

**Authors:** Dominique Rouleau, Pierre Fournier, Aline Philibert, Betty Mbengue, Alexandre Dumont

**Affiliations:** 1 Centre de Recherche du Centre Hospitalier de l’Université de Montréal (CRCHUM), 3875 St-Urbain street, Montreal, H2W 1 V1, Canada; 2 HYGEA, Dakar, Senegal; 3 Institut de Recherche pour le Développement, UMR 216 Mère et enfant face aux infections tropicales, Paris, France

## Abstract

**Background:**

Despite working in a challenging environment plagued by persistent personnel shortages, public sector midwives in Senegal play a key role in tackling maternal mortality. A better understanding of how they are experiencing their work and how it is affecting them is needed in order to better address their needs and incite them to remain in their posts. This study aims to explore their job satisfaction and its effects on their burnout, intention to quit and professional mobility.

**Methods:**

A cohort of 226 midwives from 22 hospitals across Senegal participated in this longitudinal study. Their job satisfaction was measured from December 2007 to February 2008 using a multifaceted instrument developed in West Africa. Three expected effects were measured two years later: burnout, intention to quit and turnover. Descriptive statistics were reported for the midwives who stayed and left their posts during the study period. A series of multiple regressions investigated the correlations between the nine facets of job satisfaction and each effect variable, while controlling for individual and institutional characteristics.

**Results:**

Despite nearly two thirds (58.9%) of midwives reporting the intention to quit within a year (mainly to pursue new professional training), only 9% annual turnover was found in the study (41/226 over 2 years). Departures were largely voluntary (92%) and entirely domestic. Overall the midwives reported themselves moderately satisfied; least contented with their “remuneration” and “work environment” and most satisfied with the “morale” and “job security” facets of their work. On the three dimensions of the Maslach Burnout Inventory, very high levels of emotional exhaustion (80.0%) and depersonalization (57.8%) were reported, while levels of diminished personal accomplishment were low (12.4%). Burnout was identified in more than half of the sample (55%). Experiencing emotional exhaustion was inversely associated with “remuneration” and “task” satisfaction, actively job searching was associated with being dissatisfied with job “security” and voluntary quitting was associated with dissatisfaction with “continuing education”.

**Conclusions:**

This study found that although midwives seem to be experiencing burnout and unhappiness with their working conditions, they retain a strong sense of confidence and accomplishment in their work. It also suggests that strategies to retain them in their positions and in the profession should emphasize continuing education.

## Background

Human resources are at the heart of every healthcare system as the human link between knowledge and care. However, as highlighted by the World Health Organization Report on Health 2006 [1], the healthcare workforce across the globe is facing a crisis. This is felt particularly acutely in the region of sub-Saharan Africa, which only has 3% of the global health workforce to tackle 24% of the world’s burden of morbidity [1]. Legacies of poor planning and strict economic reforms have led to a persistent shortage of qualified personnel across the region [2,3]. A brain drain of qualified health personnel, increasingly migrating to more developed countries or leaving the public sector in favour of private or non-governmental organisation (NGO) jobs [2,4,5], is further depleting the public health workforce. Regional inequalities also exist within countries, whereby health resources are concentrated in urban areas, leaving rural zones where most of the population lives even more underserved [2,6]. Health professionals who remain to work in the public system are facing heavy workloads due to the combined burden of infectious and chronic diseases, particularly related to HIV/AIDS [3,7]. The result of this situation is a workforce that is increasingly demoralised, stressed and dissatisfied.

This study focuses on Senegal, a low income francophone West African country that is suffering from a critical shortage of skilled health personnel, including those essential for maternal care: midwives, obstetricians, anaesthetists and paediatricians [8]. Senegal is facing persistently high maternal mortality ratios, (2008 estimate: 401 per 100 000 live births, CI: 252 – 622 [9]), and the lifetime risk of maternal death of 1 in 46 [8]. Despite 87% of pregnant women seeking at least one prenatal check-up, only 52% of births are assisted by a qualified professional, and that rate drops down to 33% in rural areas [10].

Midwives are a strategic component in reduction of maternal mortality in Senegal, as they attend births more frequently than other health care providers in both urban and rural areas [8]. A study carried out in the country found that hospital based midwives could detect obstetric complications, administer the necessary care and therefore expose their patients to a lesser risk of death more successfully than nurses and traditional birth attendants, and at a comparable level to doctors [11].

Senegal was highlighted as one of seven countries that needs to triple or quadruple its midwifery workforce in order to achieve the target of 95% coverage of all births with skilled birth attendants, as part of the 5^th^ millennium development goal, aimed at improving maternal health [8]. The country currently has a national average of 2 midwives per 1000 births compared to the benchmark of 6 per 1000 births set by the WHO [8]. Disparities are equally apparent in the coverage of midwives between urban and rural areas, as just over half (51%) of the midwives working in the public system are based in Dakar [12]. The capital city has 0.17 midwives per 1000 inhabitants, whereas several other regions have only 0.03 midwives per 1000 inhabitants [12].

The majority of births in Senegal take place in public health facilities, particularly in urban areas (urban: 81% public, 9% private, 10% home; and rural: 45% public, 2% private, 53% home) [8]. The private for-profit sector is largely located in Dakar and Thies, and there are approximately 70 private maternity centres and even more clinics nationally [12]. Nevertheless, most of the midwives working in Senegal are employed by the public sector as permanent civil servants or contractual labour. The proportion of midwives working in the public sector cited by one study remained consistently high (93%) between 1993 and 2000, as compared to only 50% of doctors [13].

The growing private sector in the capital is one of the probable causes of the strong pull for the urban migration of midwives, as few of them recruited to rural areas are said to remain there for long [12]. Very little is known about the dual practice activities of midwives, although evidence from other Sub Saharan African countries suggests that the practice is present [14,15] and opportunities for midwives to provide private services in clinics in the capital appear to be abundant. As elsewhere in the region, the private sector remains relatively unregulated and very little specific information about it is available.

Mechanisms to deploy and retain midwives in rural areas are being tested but the problem remains important and complex [12]. An alternate strategy employed to tackle the shortage of midwives has been the introduction of private midwifery schools in the 1990s, which have helped to steadily increase the number of nationally certified graduates from 38 in 2002 to 291 in 2008. However, there are some concerns about the quality of private midwifery education, since national exam passing rates for graduates from those schools are significantly lower than rates from publicly educated midwifery students [12]. The number of midwives recruited into the public service has also been increasing, with the exception of a hiring freeze in 2005 [12]. Information specifically on the unemployment rate for midwives in Senegal was not available.

Despite the relative scarcity of Senegalese midwives and the key role they play in the promotion of maternal and child survival in the country, relatively little is known about how they experience their work, its effects on their wellbeing and their movements within the system. This study therefore aims to explore the job satisfaction, burnout, intention to quit, turnover rates and destinations of a national sample of midwives in Senegal. Its longitudinal design also allows for an investigation of which facets of job satisfaction are associated with three purported effects: burnout, job search activities and turnover. This information could potentially help to formulate policies to improve midwives’ working conditions based on how they are experiencing their work, as well as inform initiatives to reduce attrition and address the midwife shortages in Senegal.

### Job satisfaction

Job satisfaction is a construct from the field of organisational behaviour that measures one’s attitudes towards their work [16]. It has received a great deal of attention in research because of its potential effects on the behaviours and wellbeing of professionals [17]. Job dissatisfaction has been related to a number of workplace withdrawal behaviours such as absenteeism [18] and the intention to quit [19-21], and it is the most frequently cited reason for nurse turnover [22]. In addition, research has found a link with individual health, showing that being dissatisfied with one’s job puts an individual at greater risk of burnout, anxiety and depression [23].

Nurses have been the most frequently studied group of healthcare workers, and a literature review of the principal sources of their satisfaction identified: working conditions, interactions with others, the nature of the work itself, remuneration, promotions/professional development, recognition, control/responsibility, job security and administration [24]. International studies of job satisfaction have revealed interesting variations between countries, suggesting that macro-level cultural, economic and political factors, such as labour policies and work culture and expectations, might also significantly shape an individual’s attitudes towards their work [25,26].

The job satisfaction of African healthcare workers has only recently begun to be studied and research has generally found high levels of dissatisfaction across different countries, including Kenya [27], South Africa, [28-30] Uganda [31] and others [32]. A number of qualitative and quantitative studies have highlighted the influence of certain context specific work related factors on job satisfaction, such as insufficient remuneration, poor working conditions and the HIV/AIDS epidemic [27,29,30,32,33].

Relatively few studies of job satisfaction focus specifically on midwives, despite some evidence that their professional group experiences significantly lower levels of satisfaction than doctors and nurses [34]. For the purpose of this research, a closer look will be taken at two of the many documented consequences of low job satisfaction: burnout and turnover. To our knowledge, this is the first published study to specifically investigate the job satisfaction of African midwives and its effects.

### Burnout

Burnout is a negative psychological syndrome that individuals develop in response to chronic stressors at work, which has been widely studied across different groups and countries [35]. The most popular conceptualisation of burnout comes from Maslach and Jackson, who characterize it as a reaction to an imbalance between work-related demands and personal resources, that is manifested though feelings of emotional exhaustion (EE), depersonalisation (DP) and diminished personal accomplishment (DPA) [36]. High levels of job stress, heavy workloads, the absence of social support and role ambiguity have all been reported to contribute significantly to the onset of burnout symptoms [37], which in turn have been linked to negative attitudes towards patients and detachment from one’s job (absenteeism and turnover) [38-43]. Several studies from around the world also found that working closely with people living with HIV/AIDS is an important risk factor for burnout [43-47].

Research on burnout among midwives found varying levels across different countries and work settings, but seemed to suggest that midwives are a professional group particularly at risk [48-50], emphasising the importance of autonomy, work setting (home vs. hospital) and patient demands.

The Maslach Burnout Inventory (MBI) is the most frequently used measure of burnout, having been applied in more than 90% of all empirical burnout studies up to the 1990s [51]. It consists of 3 separate sub-scales to measure each of the different components of burnout: emotional exhaustion (EE), depersonalisation (DP) and diminished personal accomplishment (DPA). Its authors report strong reliability for the MBI, with Cronbach’s alpha coefficients for the subscales of 0.90 for EE, 0.79 for DP and 0.71, consistent test-retest reliability, as well as satisfactory convergent and discriminant validity [35].

The MBI has been used extensively throughout North America and Europe, and now increasingly in developing countries. The predominance of the MBI in burnout research, along with several validated translations (including into French, German and Dutch) [36] has facilitated international research and comparisons and highlighted differences in the levels of burnout reported on the three subscales between countries [25,39,52]. International studies have generally confirmed the three factor structure of the MBI and provide evidence that the MBI is a useful tool across a wide range of occupations, languages and countries [52-55].

Several recent studies with samples of sub-Saharan African healthcare workers, most notably in, Malawi [56] South Africa [57] and Zambia [47], have noted high levels on the EE subscale, but variable levels of DP and DAP scores. Qualitative work in the region has also identified what seem to be pervasive signs of burnout resulting from the working conditions of the public healthcare system and noted its negative effects on the quality of interactions with clients [42,43,47]. However, the relationship between job satisfaction and burnout remains to be more intensely explored in the region.

### Turnover

As previously stated, job dissatisfaction has been linked to the propensity of individuals to leave their jobs. Turnover can be defined as the process whereby staff leave or transfer within the health care system [22]. It is possible to distinguish between voluntary turnover (i.e. leaving to find another job) and non-voluntary (i.e. being dismissed), as well as to differentiate turnover movement that is intra-sector (e.g. from a rural to urban public hospital), inter-sector (e.g. from public to private, or leaving the health care system altogether) and international [1]. Although turnover is a normal part of the professional life-cycle, and can be beneficial in some cases, it is the cause of concern when it happens at increasingly fast rates and in a context of pre-existing shortages and workforce imbalances [58].

A conceptual literature review by Steel in 2009 [59] identified three “core mechanisms” in the voluntary turnover process: job satisfaction, intention to quit/stay and job search mechanisms (perception of alternatives or market related measures such as unemployment rates), though the actual decision to leave one’s job also depends on wider economic and political conditions, as well as individual characteristics such as age, profession and kinship responsibilities [22].

High levels of turnover are a root cause of current shortages and mal-distributions of the health workforce, which have been associated with varying levels of health and survival across and within countries. An econometric study of 117 countries found that the density of human resources for health significantly accounted for variations in infant mortality rate, under-five mortality rate and especially maternal mortality rate, while controlling for revenue, women’s literacy and levels of absolute poverty [60].

Finally, high levels of turnover in institutions are known to have a negative impact on the working conditions of remaining staff, by increasing workloads, disrupting team cohesion and decreasing morale, thus creating a “vicious cycle” of turnover that is fuelling the crisis of human resources in health [21,22].

Nevertheless, detailed information on the magnitude of the problem globally and nationally, as well as specifics about the reasons and destinations of health worker movements, are not always available, particularly in African countries [1,3,61]. Evidence of turnover in Sub-Saharan Africa is often anecdotal, and most of the information is generally limited to registered doctors and nurses [1,62]. Due to the weakness of local human resources information, many African ministries of health rely on proxy measures to estimate the extent of their health professional emigration by gleaning data presented by destination countries, such as expatriation rates [31]. For example, Senegal has an expatriation rate of 43% for doctors, meaning that there are nearly as many doctors born in Senegal working in OECD countries (particularly France and the United States) than there are working in their home country [63]. Finally, much of the research on turnover is affected by methodological flaws, such as the use of unreliable institutional records, the predominance of transversal study designs, and the lack of specificity regarding the types of turnover studied (voluntary, non-voluntary, etc.) [1,22,64,65].

In summary, although literature on job satisfaction, burnout and turnover is well developed in western countries, and seems to have generated broad support for their interrelationships and their relevance to the human resource crisis, research has only recently begun to investigate these issues together in sub-Saharan African health workforces. The evidence available suggests that the workforce in the region is dissatisfied with their work, experiencing significant levels of burnout, in critically short supply and exiting in large numbers from the public system. Variability between countries and professional groups has underlined the importance of studying these concepts in their particular context, rather than generalizing findings. This study therefore seeks to help fill these gaps from the perspective of Senegalese midwives, in order to inform policy that reflects their needs and preferences.

Three trends are expected for the links between job satisfaction and its purported effects: job satisfaction will be negatively related with levels of burnout (Hypothesis 1), job search activities (Hypothesis 2) and turnover (Hypothesis 3) (See additional file 1 for a graphic illustration of these hypotheses in an analytical framework).

## Methods

### Variables and data collection

The present study is a secondary investigation within the larger study on maternal mortality, the QUARITE cluster-randomized trial [66], which seeks to evaluate the efficacy of the Gesta International Program in improving maternal health outcomes in both Senegal and Mali (2007–2011). Data on the individual characteristics and job satisfaction of maternity personnel were collected as part of the QUARITE study from December 2007 to February 2008 (see additional file 2 for the questionnaire). The second data collection phase on burnout, intention to quit and turnover of midwives took place during subsequent site visits over the period from December 2009 to February 2010 (questionnaire available upon request). The two-year time lapse allowed for a large turnover sample and was not expected to eliminate the associations between job satisfaction and its effects.

### Study sample

*Hospital inclusion criteria*: the 24 health facilities included in this study (from the QUARITE trial) were selected from 26 eligible public sector reference hospitals in Senegal based on four criteria: having functional operating rooms, carrying out more than 800 deliveries annually, having written consent to participate in QUARITE provided by local authorities and not having a pre-existing structured program for carrying out maternal death audits. A total of six capital, ten regional and eight district level hospitals were retained. This sample is considered representative of the variety of the contexts (urban versus rural) and of the levels of care (primary versus secondary referral health facilities) found in Senegal (See additional file 3 for a map of the hospital sites included in this study).

*Midwife inclusion criteria:* the cohort of midwives in this study included all those who were employed in the targeted public hospital maternity wards at the time of the data collection on job satisfaction (n = 235), with the exception of those who declined the invitation to participate in either phase of the investigation (T0, n = 6; T1, n = 2) and those who left more than 5% of their questions blank in the burnout questionnaire (n = 1). Midwives who left their jobs for non-voluntary reasons (death and retirement, n = 3) were excluded from the subsequent analyses involving turnover (voluntary turnover n = 38). Therefore, a total of 226 midwives were followed for the 2 years of this study, and 185 remained in their jobs to participate in the second phase.

Recruitment was made possible by collaborating with the data collection team of QUARITE and the authorities of the hospitals and maternity units, who facilitated access to the health care personnel and made the initial contact with the eligible midwives to inform them of the aims of the study and invite them to participate. In general, very high levels of participation in both phases of the investigation (T0: 229/235 (97.4%), T1: 185/188 (98.4%)) were made possible without monetary compensation because of the lasting and functional collaboration between the parties and the familiarity of the staff with the aims and procedures of the QUARITE project. One on one interviews were also made more convenient for midwives as they were done either at work or at their homes, depending on their preference.

The socio-demographic characteristics in the sample documented were: age (years), tenure (years), number of years in the profession (years), education level (middle school, high school or higher), job status (permanent civil servant or contractual), level in the professional hierarchy (head midwife/unit chief or midwife), as well as information about the individual’s perception of the availability of attractive job alternatives (unlikely, possible or certain), the type of hospital where they work (capital, regional or district) and the person doing the interview (D.R. or B.M.).

Job satisfaction can be evaluated by a variety of instruments, either global or multi-facetted, though no measurement tool is considered a “gold standard” [67]. In this study, it was measured with a multifaceted instrument developed by previous work with healthcare professionals in Mali and Burkina Faso [68,69]. An instrument composed of 42 items (with Likert scales ranging from 1 (very unhappy) to 5 (very happy)) grouped into six job facets was used for the data collection. The instrument was then restructured through several steps of content and reliability validation into a format with 29 items divided into nine job facets: “remuneration” (two items), “work environment” (five items), “workload” (four items), “tasks” (three items), “working relationships” (five items), “continuing education” (two items), “management” (four items), “morale” (two items) and “job security” (two items) (See additional file 4 for a description of the instrument). The 9 facet model showed satisfactory content validity according to criteria set out by Van Saane et al. [67].

The Maslach Burnout Inventory is considered the “gold standard” in burnout research [36]. The questionnaire [35] consists of 22 items divided into three sub-scales: emotional exhaustion (EE), depersonalisation (DP) and diminished personal accomplishment (DPA). Scores on the three burnout dimension were then categorised into “high,” “average” and “low” according to cut-off levels indicated in Maslach and Jackson’s MBI Manual [35], which are equivalent to the tertiles of a normative distribution of scores from a large sample of North American nurses and doctors. The French-language version of the MBI used in this study was translated and validated by Dion and Tessier [70] using Quebec samples of 260 day-care workers and 123 nurses. Their results gave a positive assessment of the psychometric qualities of their translation, including good internal consistency, long-range stability, factor validity and convergent validity (high correlations with measures of depression, anxiety and stress). Although the instrument has never been tested in a Francophone West African setting, evidence of its psychometric robustness, the cross-national validity of the original instrument as well as discussions with key informants during the piloting phase gave us assurances that it would appropriate for use in this study.

Midwives’ intention to quit was assessed by a question about whether they were actively searching for alternatives with three answer options: not searching, intention to search, and actively searching. To provide additional information, questions were also included about the immediacy of their intention to quit and their preferred job alternative.

Finally, turnover was measured using health personnel registries and informal interviews with remaining midwives, which included specific information about the timing and destination of the departure. Turnover was then classified as voluntary or involuntary, as well as differentiated among intra-sector (movements within the public health system), inter-sector (movements outside of the public health system) and international movements [71].

### Ethical considerations

As a complementary study included in the “satisfaction” component of the QUARITE trial, this study benefited from ethical approval by both the research ethics committee of the Sainte-Justine University Hospital Center in Montreal (renewed annually, protocol # 2425) and the national ethics committee of Senegal. Participants in the hospitals were selected on the basis of written consent provided by local authorities at the hospital level, individual signed consent at the time of the first phase of the study and confirmatory oral consent during the second phase. Eligible midwives were recruited on a voluntary basis and informed at each step of the option to withdraw from the study without consequences.

### Statistical analysis

For the data collected in phase two, missing values were replaced with the mode of the relevant questions and individuals with more than 5% of their questions unanswered were eliminated (n = 1).

### Selection of variables of interest

For burnout, and intention to quit, separate principal component analyses (PCA) were carried out to select the dimensions of each variable that best discriminate individuals amongst one another (significant discrimination along the first two components). All were found to have sufficient factor loading (≥ 0.3) and were retained for further analyses.

To prevent collinearity between the co-variables in the regressions, “number of years in the profession” was removed due to its strong correlation with “age” (Pearson’s correlation R^2^ = 0.61, *P* ≤ 0.0001). In multiple linear and logistical regressions, collinerarity was verified by variance inflation factor (VIF > 5), tolerance (> 30) indices and correlation matrices respectively.

### Descriptive statistics and multiple regressions

The socio-demographic characteristics of the midwives who left and those who stayed were compared using ANOVAs or Chi-square tests (likelihood ratio). Since age and years in current position variables failed to normality, non-parametric ANOVAs (Wilcoxon/Kruskall-Wallis test) were used.

To assess the relationship between burnout (response variable) and job satisfaction (explanatory variable), a series of multiple linear regressions were performed between each dimension of burnout and facets of job satisfaction separately (3*9 =27). These regressions were controlled by the following co-variables: age, tenure, type of institution, educational attainment, rank, employee status and interviewer. For each burnout dimension, the job satisfaction facets that showed a *P* value less than 0.10 were then entered simultaneously into a single multiple regression controlling for the same co-variables.

A similar two-step procedure was performed between job search, turnover (response variables) and job satisfaction (explanatory variable), using a multiple logistical regression. The controlling variables were the same as above, except that “perception of alternatives” was also controlled in the regressions with job search.

To allow for logistical regressions, the job satisfaction facet variables were categorised into the lower 25^th^ percentile (least satisfied) versus the rest. Similarly, the three possible answer options of the job search activities item were merged into two modalities, in accordance with their meaning: not searching or intent to search versus actively searching.

The two-step procedure for the multiple regressions was chosen because of the exploratory aims of the study. In all analyses, outliers were eliminated on the basis of the Cook’s D Influence test (coefficient greater than 0.2).

All analyses were performed using SPSS 17 and/or JMP7 statistical software, and with a significance level of alpha = 0.05.

## Results

### Descriptive data

#### *Study sample characteristics*

In the initial sample (T0), the mean age was 40 years, ranging from 24 to 61 years old. The number of years in their current position spanned from three to 32 years, and the most frequent level of instruction was the equivalent of a high school diploma (45.1%). Seventy-one percent of the midwives were civil servants with permanent positions, and about a fifth (20.8%) had some kind of rank distinction, such as being head-midwife.

#### *Turnover*

Forty-one midwives left their jobs between December 2007 and December 2009, creating a turnover rate of 18.1% over two years (or approximately 9% annually). There were no significant differences in socio-demographic characteristics between those who left and those who stayed (Table 1).

**Table 1:** Socio-demographic characteristics of the midwives (over entire study period from 2007–10)

**Variable**		**Initial sample T0 (n = 226)**	**Departures (n = 41)**	**Remaining midwives T1 (n = 185)**	**P value ( *P* ** **< 0.05)**
		**Mean (SD)**	**Mean (SD)**	**Mean (SD)**	**p**
**Age (years)**		40.4 (9.2)	40.9 (9.4)	40.3 (9.2)	0.71
**Years in current position (years)**		7.8 (5.7)	6.5 (4.9)	8.1 (5.9)	0.09
		**N (%)**	**N (%)**	**N (%)**	**P**
**Type of institution**	Capital	97 (42.9)	15 (36.6)	82 (44.3)	0.83
	Regional	98 (43.4)	20 (48.8)	78 (42.2)	
	District	31 (13.7)	6 (14.6)	25 (13.5)	
**Level of education**	Middle school	40 (17.7)	6 (14.6)	34 (18.4)	0.23
	High school	102 (45.1)	16 (39.0)	86 (46.5)	
	Higher	84 (37.2)	19 (46.3)	65 (35.1)	
**Rank**	Superior rank	47 (20.8)	6 (14.6)	41 (22.2)	0.22
	Midwife	179 (79.2)	35 (85.4)	144 (77.8)	
**Employee status**	Civil servant	161 (71.2)	33 (80.5)	128 (69.2)	0.08
	Contractual	65 (28.8)	8 (19.5)	57 (30.8)	

The turnover was largely voluntary (92.7%), with 28 midwives transferring to a new health facility and 10 leaving to do different vocational training. Only three midwives left for non-voluntary reasons (1 died and 2 retired). The average turnover rate for the 22 structures was 22.3%, and the highest institutional turnover was found at Goudiry (1/1), Matlaboul Fauzeni (4/8) and Ziguinchor (4/11). No turnover (0%) occurred in seven health facilities.

#### *Job satisfaction and burnout*

Overall, midwives reported themselves to be most satisfied on the facets “morale” and “job security” and least satisfied on the facets “work environment” and “remuneration.”

The results in table 2 cluster in two groups: the facets with average scores above 70/100 and those with average scores below 56/100, with “workload” in between.

**Table 2:** Summary of job satisfaction facet scores

**Rank (most to least satisfied)**	**Job facets**	Mean score/100*	**SD**
**1**	Moral satisfaction	78.36	10.88
**2**	Security	76.74	13.78
**3**	Tasks	73.45	12.86
**4**	Working relations	73.16	11.18
**5**	Workload	62.78	13.09
**6**	Continuing education	55.00	20.36
**7**	Management	53.67	15.09
**8**	Remuneration	52.35	13.83
**9**	Work environment	50.84	15.86

*0 = completely dissatisfied, 100 = completely satisfied.

Table 3 illustrates the mean burnout score and the proportion reporting levels categorised as “high” for each sub-scale, as well as the norms put forth in the MBI Manual for the occupational category “medicine” [35].

**Table 3:** Table of burnout scores

**Burnout dimension**	**Study sample (n = 185)**		**MBI manual norms**	
	**Mean Score (SD)**	**% in “high” burnout range**	**Mean Score (SD)**	**% in “high” burnout range**
**Emotional exhaustion**	35.4 (9.6)	80.0	22.2 (9.5)	33.3
**Depersonalization**	11.4 (6.1)	57.8	7.1 (5.2)	33.3
**Personal accomplishment**	39.7 (4.8)	12.4	36.5 (7.3)	33.3

“High” levels on all three sub-scales were found for 14 midwives, representing 7.57% of the study sample at T1. (See additional file 5 for the full burnout results).

#### *Intention to quit*

Among the midwives remaining at T1, more than a third (37.3%) reported to be actively looking for work elsewhere, either by independently applying for jobs, having submitted a request for transfer, or applying to return to school. A slightly larger proportion (43.2%) reported they had the intention to do so, but had not acted on it yet, and the remainder was not interested in looking (19.5%). Only 41.1% of the midwives questioned had the intention of remaining in their current position for the next several years, compared to a similar proportion (41.6%) that said they would probably try to leave before the end of the year and 17.3% who said they wanted to leave right away.

More than half (54.1%) of the midwives chose the option “do additional training” as their desired job alternative, in favour of the options “work for an NGO” (18.4%), “other” (18.4%; which was often transfers, retirement, etc.), “work in another country” (5.4%), and “work in a private clinic” (3.8%).

#### *Multivariate analyses*

The results of the analyses are described below; tables 4 and 5 present a summary of the significant associations in both types of regressions, and Additional file 6, Additional file 7, Additional file 8, Additional file 9, Additional file 10 contain tables with all the results.

**Table 4:** Summary of significant results of the linear regressions between job satisfaction and burnout

**Variables Dependent**	**Significant independent**	**1**^**st**^**Analysis**^**a**^** *P* ** **≤ 0.10**	**2**^**nd**^**Analysis**^**b**^** *P* ** **≤ 0.05**
**Emotional exhaustionScore**	**Job satisfaction facet score**		
	1. **Remuneration**	0.01	0.02
	3. Workload	0.02	0.63
	4. **Tasks**	0.01	0.03
	6. Continuing education	0.03	0.28
	7. Management	0.06	0.83
**DepersonalisationScore**	**Job satisfaction facet score**		
	2. Work environment		
	3. Workload		
	4. Tasks	0.10	0.55
	6. Continuing education	0.02	0.24
	7. Management	0.01	0.17

Controlling for: age, tenure, type of institution, educational attainment, rank, employee status and interviewer.

a: multiple regressions performed between each dimension of burnout and facet of job satisfaction separately (*P* < 0.10); b: for each burnout dimension, all significant job facets from 1^st^ analysis entered simultaneously in second multiple regression (*P* < 0.05).

**Table 5:** Summary of significant results of the logistical regressions between job satisfaction and job search and turnover

**Variables dependent**	**Significant independent**	**1**^**st**^**Analysis**^**d**^** *P* ** **≤ 0.10**	**2**^**nd**^**Analysis**^**e**^**OR (95%CI)**
Job search*^a^	**Job satisfaction facets**^**c**^		
	1. Remuneration	0.06	0.41 (0.15–1.11)
	9. **Job security**	0.00	0.16 (0.04–0.67)
Turnover**^b^	**Job satisfaction facets**		
	6. **Continuing education**	0.00	0.13 (0.03–0.44)
	7. Management	0.04	1.07 (0.29–3.92)

*Controlling for: age, tenure, type of institution, educational attainment, rank, employee status, interviewer and perception of alternatives).

** Controlling for: age, tenure, type of institution, educational attainment, rank and employee status).

Categories: a: not searching (1) vs actively searching (2), b: stayed (1) vs. quit (2), c: most dissatisfied 25 % (1) vs. rest (2).

d: multiple regressions performed between each dimension of job search/turnover and facet of job satisfaction separately (*P* < 0.10); e: for both job search and turnover, all significant job facets from 1^st^ analysis entered simultaneously in second multiple regression (*P* < 0.05).

##### *Job satisfaction and Emotional exhaustion*

Multiple linear regressions showed that 5 facets of job satisfaction were significantly (*P* ≤ 0.10) and negatively correlated to levels of EE: “remuneration”, “workload”, “tasks”, “continuing education” and “management”, but only “remuneration” (−) and “tasks” (−) remained significant at *P* ≤ 0.05 in the second combined regression.

##### *Job satisfaction and Depersonalisation*

Multiple linear regressions showed that levels of satisfaction on 5 job facets were significantly (*P* ≤ 0.10) and negatively correlated to levels of DP: “work environment,” “workload,” “tasks,” “continuing education” and “management,” but none remained significantly associated in the second combined regression (*P* ≤ 0.05).

##### *Job satisfaction and Diminished Personal Accomplishment*

No facets of job satisfaction were found to be significantly correlated to levels of DPA in step one (*P* ≤ 0.10), and therefore step two was omitted.

##### *Job satisfaction and job search*

Logistical regressions showed being dissatisfied on the job facets “remuneration” and “job security” was significantly (*P* ≤ 0.10) and *negatively* correlated with actively searching for work. Only “job security” remained (−) significant in the second round (*P* ≤ 0.05). The odds of being actively searching for alternative jobs is 6.2 times higher for the 25% of midwives most dissatisfied with their job security, compared to the rest.

##### *Job satisfaction and turnover*

Logistical regressions showed that being dissatisfied on the facets “continuing education” and “management” was significantly (*P* ≤ 0.10) and *negatively* correlated with the act of staying in one’s job. When these two significant facets were analysed together, only “continuing education” remained significantly associated with turnover (*P* ≤ 0.05). The odds of actually quitting one’s job is 8.4 times higher for the midwives most dissatisfied with their opportunities for continuing education, compared to the rest.

## Discussion

This study allowed a close look at the professional lives of hospital midwives across Senegal, and uncovered some findings consistent with health care workers in similar environments, and others that appear to be specific to the midwifery profession. The longitudinal design of this study reduced the causal ambiguity associated with cross-sectional analyses of the associations between job satisfactions and its effects. In addition, the considerable proportion of midwives practicing in surgical maternities in the Senegalese public health system in 2008 included in our sample (226/340 or 66.4% [72]), along with the high response rates in both phases (97.4% and 98.4%, respectively), support the potential representativeness of our results for midwives across the country.

Midwives were found to be generally dissatisfied with aspects of their work related to their working conditions, and to be experiencing certain aspects of burnout (exhaustion and depersonalisation), but not others (diminished personal accomplishment). The turnover rate over two years was low considering the pervasive intention to quit, and pursuing a new professional specialty was found to be a frequently sought after alternative job destination. It was also notable that job searching activities and actual turnover were significantly correlated to different job satisfaction facets. These and other findings are discussed in detail below.

Job satisfaction was assessed in this study using a multi-dimensional instrument adapted to the West African healthcare setting. The cluster of job facets with which midwives were relatively dissatisfied (“continuing education”, “management”, “remuneration” and “work environment” (which could also include “workload”)) is consistent with other studies in the sub-Saharan African region that identified the most important sources of discontentment as being related to the limited resources of health care systems (financial, material and other) [27,29,30,33]. The material environment of Senegalese public hospitals has been described as insufficient and ill-adapted to the skills of many health professionals [12]. In addition, a sentiment of discontentment amongst health workers in Senegal has led to frequent strikes, for which the main grievances relate to the work environments, financial management and salaries [73-76]. Salaries for midwives in Senegal are likely to be lower than their expectations due to the escalating cost of living and because most have large extended families to provide for (unpublished data from the QUARITE trial).

On the other hand, the cluster of job facets with which midwives were relatively satisfied: “morale”, “security”, “tasks” and “working relations”, seems to be more closely related to the social and professional aspects of midwifery work [77]. Of the nine facets studied, midwives reported themselves happiest with their job “morale”; a facet composed of two items that specifically measure happiness with the “quality of the work they do” and “the outcomes of the births and health of the mothers and infants.” This tendency could be due to the relatively straightforward and specific nature of their work: it has a clear outcome that is often positive (as opposed, for instance, to managing a chronic disease). The technical specificity of midwifery is likely to have contributed to feelings that they have mastered their professional skills and have confidence in their abilities [49]. On the other hand, the reported happiness with job security is likely due to the fact that almost three-quarters of the midwives in the present study are civil servants.

### Burned-out midwives

An important finding of this study was the very large proportion of midwives that were found to have levels of emotional exhaustion classified as “high” (80%) and as “average–high” (94%). This prevalence of EE exceeds levels found in other studies of sub-Saharan health care workers, such as mixed samples of professionals from Zambia [47], Malawi [57] and South Africa [78], in which “average-high” EE levels are found in 62, 66 and 69% of the samples, respectively. Scores on the depersonalisation subscale were also comparatively high, but closer to average levels reported by other studies with African health care workers [56,57] and to MBI norms [35].

The high levels of EE in this study could potentially be explained by the nature of the midwifery profession, as previous studies have identified midwives as an occupational group at risk of experiencing EE and burnout [48,49]. According to Maslach and Jackson [35], working with service recipients who are “experiencing distress, pain or anger” is an important precursor for the development of EE, and Senegalese midwives’ work inherently involves close contact with mothers who suffer acute birth pains, often without analgesics. The important “emotional content” of midwifery work has been noted [79], and this has also identified as a contributing factor for developing burnout [80]. Finally, EE is the dimension of burnout that most closely resembles a traditional measure of stress, [81] and many aspects of their hospital working conditions are highly stressful, such as the long shift durations (often 12–24 h), and having to deal with nervous and impatient family members in addition to patient demands and heavy workloads.

Conversely, the large proportion of “high” scores found on the personal accomplishment scale (51.9%) is a sign of *low* burnout, which goes against the expected trend. This indicates that stressful working conditions do not seem to negatively affect midwives’ self-evaluated sense of competence and achievement at work. It is likely that this is related to the high levels of work morale (with respect to quality and outcomes), and thus also potentially related to the technical specificity midwifery work.

Alternatively, it has been suggested that cultural factors can affect the experience of burnout, particularly in settings that differ from the typical individualistic American work culture, in which work takes on an “existential meaning” and occupational stress affects one’s self-conception [36,52,82,83]. Although the effects of cultural variations on the measurement of burnout need to be further explored, it is possible that the expectations and experience of work in Senegal contributed to the inversely high levels of personal accomplishment found in this study.

Finally, it must be highlighted that when the conventional criteria for burnout are used, only 7.6% of the sample is considered “burned-out” (whereby a person is considered to be burned-out if he or she scores within the “high” range on all 3 dimensions of the MBI [35]). However, some scholars now recommend using revised criteria for assessing burnout with the MBI [84,85]. This is due to a growing consensus within burnout research that the PA dimension is a “marginal” part of the burnout syndrome [86], and possibly a “coping mechanism” that develops independently from the other two core dimensions of burnout [41,87,88]. The alternative criteria thus consider burnout to be a “high” EE score, combined with a “high” score in either DP or DPA. When those criteria are applied, the prevalence of severe burnout increases more than sevenfold, from 7 to 55.7%. The results in this study tend to support employing the latter criteria, because of the particularly high levels found on what are considered the two “core” dimensions of burnout: EE and DP.

### Midwife mobility

The overall turnover rate calculated in this study is 18.1% over two years, or 9% annually. This is significantly lower than attrition rates in British or American hospitals, which are usually in the range of 15-20% for nurses [64,89,90]. When we compare our results to a rare study that reports turnover rates of hospital staff in the West Nile region of Uganda, our 9% turnover rate appears high and several similarities are notable. The study by Onzubo [91] looked at the attrition rates in three Government General Hospitals over a period of five years using staff records and interviews, and found attrition rates of 4%, 3% and 3% respectively. The data also showed that the average annual attrition rate for government employees was highest among the medical officers (19%), followed by the enrolled midwives (5%). The most frequent destination was transfers to other government institutions (45.2%) and lower level health units (16.1%), and they also found that limited access to transfers between placements in one district to another was a reason given for long service in government hospitals (identified as the second most frequent reason (16%)). It is unclear why our rates are higher than those found in this Ugandan study, though it could be due to their smaller hospital sample size.

Nevertheless, the permanent losses to the midwife workforce (not including transfers within the public health system) occurred at an average annual rate of only 2.9% (13/226 over 2 years). This is lower than the WHO estimated average attrition rate of of 5% per year for doctors, nurses and midwives applied in 13 sub-Saharan countries (due to mortality, international migration and retirement) [61].

It is notable that the observed turnover level is relatively low compared to the widespread intent to quit and active job search measured in the sample at T2. This is likely due to the fact that Senegalese midwives do not have many opportunities to act on their intention to leave, which is related to the rigidity of the labour market for midwives in the country. The labour market rigidity is caused by the near employment monopoly of the public sector (where 93% of them work [13]), which dictates the terms of employment (e.g. recruitment and contracts) and controls inter-public sector transfers. Limitations on transfers within the public system were also highlighted by the study in Uganda by Onzubo [91] as a factor influencing the length of service. In addition, the specific nature of the midwifery professional skill set might also limit their alternative employment opportunities as compared to nurses, for example [13]. It is more likely in this case that midwives moonlight – or take on a second job outside of the hospital where they work, rather than quit. This is a frequent coping behaviour seen among healthcare workers in Africa who express dissatisfaction with their remuneration and working conditions but have few job alternatives [14,98].

It is interesting to highlight that no international migration was documented during the two years of the study, which indicates the clear preference of midwives for intra-national mobility. In fact, international migration was one of the least desired alternatives to their current job (only 5.6% of respondents chose that option). It is possible that this is because of issues of certification equivalency or the competitiveness of the midwife job market in other francophone countries, though it could also be due to the fact that midwives are exclusively female and thus likely to have families and kinship responsibilities that would limit their ability to move abroad for work.

In addition, it is notable that the majority (52%) of midwives chose the option to “do additional training” as their desired alternative work activity, and more than one third of the voluntary turnover cases were of midwives returning to school to become trained as a “superior technician.” This highlights an unexpectedly strong preference for inter-sector movement – or leaving the profession [7], which is significant because that type of departure is a permanent loss to the midwifery workforce. This is not a trend frequently observed in the region [7] and the reasons motivating it should be explored further.

### The effects of job satisfaction

Results from the multivariate analyses between job satisfaction and burnout are consistent with other studies that found a strong link between workplace dissatisfaction and both EE and DP [23,93-96]. The lack of significant association between all the job satisfaction facets and PA goes against the predicted outcome, though similar results have been shown elsewhere [55]. These results therefore partially verify hypothesis 1 (a negative relationship between JS and BT), though comparisons with previous research relating job satisfaction to burnout are limited as most studies use cross-sectional designs and global rather than multi-facetted instruments to measure job satisfaction.

It is notable that while verifying hypotheses 2 and 3, the analyses relating job satisfaction to active job search and turnover found different job facets to be significantly associated with each variable: midwives were found to be more likely to be actively looking for work if they were among the most dissatisfied with their job security, whereas the act of leaving one’s job was most strongly related to being dissatisfied with one’s opportunities for continuing education. This seems to support the earlier postulate that financial considerations such as job insecurity drive midwives to seek alternatives, but that limited opportunities outside the public sector restrain their ultimate movement. Furthermore, the link found between dissatisfaction with continuing education and actual turnover is also in line with the tendency observed in this study for midwives to leave their profession and pursue new vocational training, which could be a means for them to satisfy their desire for professional stimulation and advancement. It is important that such career and knowledge related concerns were more significant predictors of actually leaving than the financial ones because dissatisfaction with remuneration is so widespread.

### Limitations of the study

The very high levels of EE warrant caution. It is possible that they were influenced by a similar reporting bias as noted by Kruse, Chapula et al.[43] in their study of burnout in Zambia, in which the healthcare professionals interviewed were potentially tempted to “exaggerate” experienced exhaustion to try to influence policy. In addition, it is possible that the open ended questions related to stress and the introductory definition of burnout that preceded the administration of the burnout questionnaire might have also led midwives to overstate their levels of stress. This was nevertheless kept as part of the interview process, because during the pilot phase of the instrument, key informants suggested that unfamiliarity with the concept of burnout would make presenting the objectives of the study and administrating the questions more difficult. Finally, the MBI is not validated in Senegal and the interpretation of the burnout results must be done with caution due to its reliance on North American based cut-off points. Although several studies have used the MBI in Africa, none of these offered a critical analysis of the instrument’s cross-cultural validity in an African setting.

## Conclusions

This study offers a unique portrait of how Senegalese midwives experience their work. They were found to be dissatisfied with their working conditions and remuneration and to be suffering from extremely high levels of emotional exhaustion and high levels of depersonalisation, which are likely to be related to the long working hours, heavy workloads and challenging working conditions in which they practice midwifery. These observations are very worrying and should be further explored, as elevated levels of discontentment and exhaustion at work could have potentially serious impacts on their physical health and the quality of care they give their patients.

In contrast, these challenges seem to have relatively unaffected the sense of accomplishment and confidence they feel at work, thus indicating that midwives are resilient and remain pleased with important professional aspects of their job. The combination of widespread discontentment and exhaustion with high work-esteem may be suggestive of an experience of work that differs from the “existential” work-centered professional culture around which the MBI was developed in the West, and perhaps the necessity of measuring burnout in the region with an alternative instrument developed locally.

And finally, it is encouraging that midwives seem to be unlikely to leave the country for work and thus not significantly contributing to the Senegalese “brain drain.” However, the reasons behind the unexpectedly high levels of interest in and effective departures from the midwifery profession should be further explored. It seems that the most professionally mobile midwives tend to be particularly ambitious and motivated, and so policies and interventions aiming to retain them should emphasise continuing education and professional opportunities within the profession. This has interesting implications for the QUARITE trial, because the GESTA International program includes aspects of continued education and training, and could therefore lead to the dual and mutually reinforcing benefits of improving emergency obstetric care while reducing turnover.

## Abbreviations

AIDS, Acquired Immunodeficiency Syndrome; CI, Confidence interval; DP, Depersonalisation; EE, Emotional Exhaustion; GESTA, Gestion du travail et de l’accouchement (maternal health program); HIV, Human Immunodeficiency Virus; MBI, Maslach Burnout Inventory; NGO, Non-governmental organization; OECD, Organisation for Economic Co-operation and Development; OR, Odds ratio; PA/DPA, Personal Accomplishment/Diminished Personal Accomplishment; QUARITE, Qualité des soins gestion du risque et techniques obstétricales; SD, Standard Deviation.

## Competing interests

The authors declare that they have no competing interests.

## Authors’ contributions

DR conceived of the study, carried out the data collection in phase two, performed the statistical analyses, and drafted and edited the article. PF contributed to the conception and design of the study, the interpretation of the data and the revision of the article. AP assisted with the statistical analyses and the editing of the article. BM was a significant contributor to the acquisition of the data, and helped to carry out the data collections of phases one and two. AD contributed to the design and coordination of the study, facilitated the acquisition of the data from phase one and contributed to reviewing the article. All authors read and approved the final manuscript.

## Supplementary Material

Additional file 1 Analytical framework of the study and research. Click here for file

Additional file 2 Phase 1 questionnaire on job satisfaction. Click here for file

Additional file 3 Map of the targeted hospital sites (.pdf) (Source of the original map: http://www.afrique-planete.com). Click here for file

Additional file 4 Description of the job satisfaction instrument. Click here for file

Additional file 5 Complete table of burnout ranges of the study sample. Click here for file

Additional file 6 Complete results from the 2-step linear regressions analyses of job satisfaction scores (independent) and emotional exhaustion scores (dependent). Click here for file

Additional file 7 Complete results from the 2-step linear regressions analyses of job satisfaction scores (independent) and depersonalisation scores (dependent). Click here for file

Additional file 8 Complete results from the 2-step linear regressions analyses of job satisfaction scores (independent) and personal accomplishment score (dependent). Click here for file

Additional file 9 Complete results from the 2-step logistical regressions analyses of job satisfaction (independent) and job search (dependent). Click here for file

Additional file 10 Complete results from the 2-step logistical regressions analyses of job satisfaction (independent) and turnover (dependent). Click here for file
