# Supplementary material for: The effects of midwives’ job satisfaction on burnout, intention to quit and turnover: a longitudinal study in Senegal
Source: Hum Resour Health. 2012 Apr 30;10:9. doi: 10.1186/1478-4491-10-9 (PMC3444355; doi:10.1186/1478-4491-10-9)
Supplement: Additional file 1 — Analytical framework of the study and research. [file 1478-4491-10-9-S1.pdf]

Additional file 1: Analytical framework of the study and research hypotheses

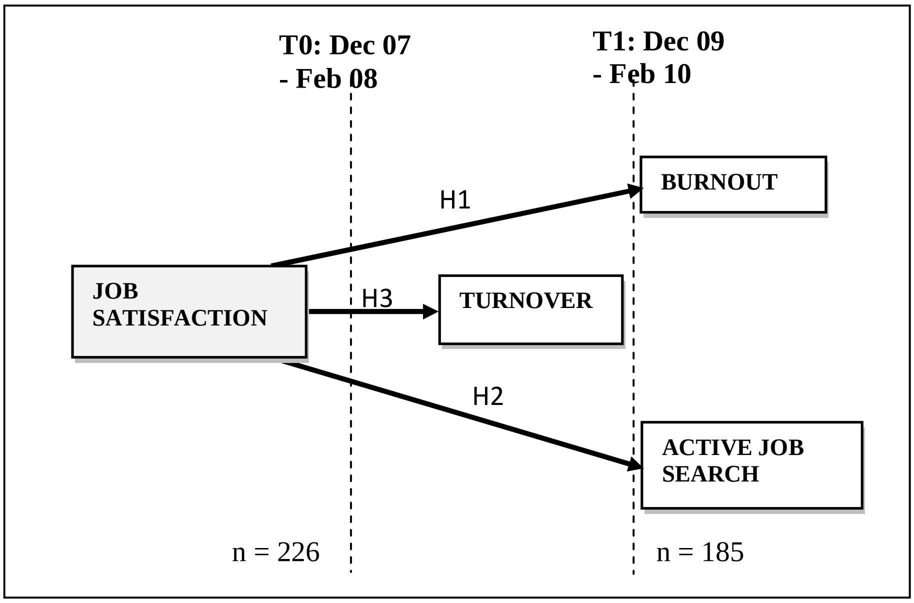

H1: Job satisfaction facet scores are negatively correlated with levels of the 3 dimensions of burnout.

H2: Job satisfaction facet scores are negatively correlated with job search activities.

H3: Job satisfaction facet scores are negatively correlated with the incidence of turnover.
