# Supplementary material for: The effects of midwives’ job satisfaction on burnout, intention to quit and turnover: a longitudinal study in Senegal
Source: Hum Resour Health. 2012 Apr 30;10:9. doi: 10.1186/1478-4491-10-9 (PMC3444355; doi:10.1186/1478-4491-10-9)
Supplement: Additional file 2 — Phase 1 questionnaire on job satisfaction. [file 1478-4491-10-9-S2.pdf]

Questionnaire - Satisfaction professionnelle

**Date : ____________________**	/__/
**Nom de l'établissement : ____**	/__//__/
**Nom de l'enquêteur : ____**	/__/
**Code du répondant : /__/__/__/__/__/** (Rappel : anonymat)	/_/_/_/_/_/
**Sexe :** 1 Masculin 2 Féminin	/__/
**Profession :**	/__/__/
1 Gynécologue-obstétricien *(si interne ou CES : cocher ci-dessous)*	
2 Médecin compétent en SONU	
3 Médecin généraliste non formé en SONU	
4 Pédiatre	
5 Médecin anesthésiste	
6 Interne	
7 CES	
8 Sage-femme	
9 Infirmier(e) diplômé(e) / technicien(ne) sup. à la maternité	
10 Infirmier(e) / technicien(ne) sup. anesthésiste	
11 Instrumentiste	
12 Aide opérateur	
**Niveau d'instruction :**	/__/
1 Non alphabétisé	
2 Primaire	
3 Secondaire – BFEM ou diplôme de même niveau	
4 Secondaire – Baccalauréat ou diplôme de même niveau	
5 Supérieur	
**Occupez-vous un poste de cadre ?** 1 Oui 2 Non	/__/
**Si oui, lequel :**	/__/
1 Chef de service	
2 Professeur d'université	
3 Infirmier-chef	
4 Maîtresse sage-femme	
5 Chef d'unité de soins	
6 Autre : ____________________________________	

Âge : ________ ans /__/__/

Ancienneté dans le *poste actuel* :_______ années /__/__/

Ancienneté dans la *profession* :________ années /__/__/

Statut Professionnel : /__/

1 Fonctionnaire
2 Militaire hors cadre
3 Contractuel Etat
4 Contractuel hôpital
5 Contractuel commune
6 Bénévole communautaire

Travail *pour la maternité* : 1 À temps plein 2 À temps partiel /__/

Affiliation syndicale : 1 Syndiqué(e) 2 Non syndiqué(e) /__/

Situation matrimoniale : /__/

1 Marié - Polygame
2 Marié - Monogame
3 Célibataire
4 Veuf (ve)
5 Divorcé(e)

Combien de personnes prenez-vous en charge avec votre salaire[1] ?
(en comptant vous-même, enfants, parents, domestique…) : _______ /__/__/

Habitez-vous loin de votre famille à cause de votre travail ?
1 Oui 2 Non /__/

Temps de trajet entre domicile et lieu de travail :
Aller : _____ h _____min. /_/_/_/_/
Retour : _____ h _____min. /_/_/_/_/

Moyen de transport principal : /__/

1 Voiture personnelle
2 Moto, mobylette
3 Transport en commun
4 Charrette
5 Vélo
6 À pied

---

[1] Dire le nombre de personnes pour qui vous faites des dépenses **régulières** et **assez importantes.**

Instructions : Nous allons vous poser une liste de questions sur votre satisfaction dans votre travail.
Les questions portent sur 6 aspects : la rémunération; les outils et le cadre de travail; l'organisation et le contenu du travail; la formation et la supervision; la satisfaction morale ; le style de management.
Pour chaque question, il y a 5 choix de réponse (Donner la feuille avec les choix de réponse).
Il n'y a pas de réponse vraie ou fausse : ce qui compte c'est que vous exprimiez votre opinion.

Questions sur la rémunération

**1**	Êtes-vous content(e) du montant de votre salaire ?	5<br>Très content	4<br>Content	3 Moyennement content	2<br>Mécontent	1<br>Très mécontent
**2**	Êtes-vous content(e) de la régularité de versement de votre salaire (salaire versé à temps, en retard…) ?	5<br>Très content	4<br>Content	3 Moyennement content	2<br>Mécontent	1<br>Très mécontent
**3**	Êtes-vous content(e) des primes et indemnités que vous recevez ?	5<br>Très content	4<br>Content	3 Moyennement content	2<br>Mécontent	1<br>Très mécontent
**4**	Êtes-vous content(e) des avantages sociaux dont vous bénéficiez (par ex. logement de fonction, soins de santé gratuits, colonies de vacances…) ?	5<br>Très content	4<br>Content	3 Moyennement content	2<br>Mécontent	1<br>Très mécontent
**5**	Êtes-vous content(e) de vos possibilités d'avancement dans votre corps professionnel ?	5<br>Très content	4<br>Content	3 Moyennement content	2<br>Mécontent	1<br>Très mécontent
**6**	Êtes-vous content(e) de votre stabilité d'emploi (certitude ou incertitude sur le fait de garder / perdre votre emploi) ?	5<br>Très content	4<br>Content	3 Moyennement content	2<br>Mécontent	1<br>Très mécontent

Questions sur les outils et le cadre de travail

**7**	Êtes-vous content(e) du matériel / équipement dont vous disposez pour faire votre travail ?	5<br>Très content	4<br>Content	3 Moyennement content	2<br>Mécontent	1<br>Très mécontent
**8**	Êtes-vous content(e) des locaux de travail (par ex : espace, propreté, aération, lumière, bruit, confort…) ?	5<br>Très content	4<br>Content	3 Moyennement content	2<br>Mécontent	1<br>Très mécontent
**9**	Êtes-vous content(e) de la disponibilité du sang pour les transfusions ?	5<br>Très content	4<br>Content	3 Moyennement content	2<br>Mécontent	1<br>Très mécontent
**10**	Êtes-vous content(e) des médicaments dont vous disposez pour faire votre travail ?	5<br>Très content	4<br>Content	3 Moyennement content	2<br>Mécontent	1<br>Très mécontent
**11**	Êtes-vous content(e) des consommables (par ex : coton, alcool…) dont vous disposez pour faire votre travail ?	5<br>Très content	4<br>Content	3 Moyennement content	2<br>Mécontent	1<br>Très mécontent
**12**	Êtes-vous content(e) de la protection contre les risques professionnels (par ex, contre l'exposition au VIH, contre d'autres risques) ?	5<br>Très content	4<br>Content	3 Moyennement content	2<br>Mécontent	1<br>Très mécontent
**13**	Êtes-vous content(e) des imprimés dont vous disposez pour faire votre travail (par ex, ordonnancier, bulletins d'analyses, dossier médical, partographe …) ?	5<br>Très content	4<br>Content	3 Moyennement content	2<br>Mécontent	1<br>Très mécontent
**14**	Êtes-vous content(e) des protocoles dont vous disposez pour faire votre travail ?	5<br>Très content	4<br>Content	3 Moyennement content	2<br>Mécontent	1<br>Très mécontent

Questions sur l'organisation et le contenu du travail

**15**	Êtes-vous content(e) de vos horaires de travail ?	5<br>Très content	4<br>Content	3 Moyennement content	2<br>Mécontent	1<br>Très mécontent
**16**	Êtes-vous content(e) de votre charge de travail (quantité de travail, débordé ou pas) ?	5<br>Très content	4<br>Content	3 Moyennement content	2<br>Mécontent	1<br>Très mécontent
**17**	Êtes-vous content(e) de la répartition de la charge de travail entre les membres de votre équipe ?	5<br>Très content	4<br>Content	3 Moyennement content	2<br>Mécontent	1<br>Très mécontent
**18**	Êtes-vous content(e) de l'entente qui existe entre les membres de votre service ?	5<br>Très content	4<br>Content	3 Moyennement content	2<br>Mécontent	1<br>Très mécontent
**19**	Êtes-vous content(e) de la variété de vos tâches (tâches de différentes sortes) ?	5<br>Très content	4<br>Content	3 Moyennement content	2<br>Mécontent	1<br>Très mécontent
**20**	Êtes-vous content(e) de la répartition de votre temps de travail entre les soins et vos autres tâches?	5<br>Très content	4<br>Content	3 Moyennement content	2<br>Mécontent	1<br>Très mécontent
**21**	Êtes-vous content(e) de l'adéquation qui existe entre vos tâches et vos compétences (est-ce que vos tâches correspondent bien à vos compétences) ?	5<br>Très content	4<br>Content	3 Moyennement content	2<br>Mécontent	1<br>Très mécontent
**22**	Êtes-vous content(e) du niveau de responsabilité professionnelle qui vous est confié ?	5<br>Très content	4<br>Content	3 Moyennement content	2<br>Mécontent	1<br>Très mécontent
**23**	Êtes-vous content(e) de la collaboration avec les autres services de l'hôpital (par exemple, collaboration entre maternité et laboratoire, collaboration avec la pharmacie centrale…) ?	5<br>Très content	4<br>Content	3 Moyennement content	2<br>Mécontent	1<br>Très mécontent

Questions sur la formation et la supervision

**24**	Êtes-vous content(e) de la façon dont votre formation ***initiale*** (théorique et pratique) vous a préparé à répondre aux réalités du terrain ?	5<br>Très content	4<br>Content	3 Moyennement content	2<br>Mécontent	1<br>Très mécontent
**25**	Êtes-vous content(e) de la formation que vous ***continuez*** à recevoir ?	5<br>Très content	4<br>Content	3 Moyennement content	2<br>Mécontent	1<br>Très mécontent
**26**	Êtes-vous content(e) de la façon dont on sélectionne les membres du service pour participer aux activités de formation ?	5<br>Très content	4<br>Content	3 Moyennement content	2<br>Mécontent	1<br>Très mécontent
**27**	Êtes-vous content(e) de l'appui technique (conseils, encadrement) que vous donnent vos supérieurs hiérarchiques ?	5<br>Très content	4<br>Content	3 Moyennement content	2<br>Mécontent	1<br>Très mécontent

Questions sur la satisfaction morale

**28**	Êtes-vous content(e) de la réputation, l'image de votre établissement auprès de la population ?	5<br>Très content	4<br>Content	3 Moyennement content	2<br>Mécontent	1<br>Très mécontent
**29**	Êtes-vous content(e) de la qualité des soins offerts aux patientes dans votre service ?	5<br>Très content	4<br>Content	3 Moyennement content	2<br>Mécontent	1<br>Très mécontent
**30**	Êtes-vous content(e) de l'issue des accouchements dans votre service (santé des femmes et des nouveau-nés) ?	5<br>Très content	4<br>Content	3 Moyennement content	2<br>Mécontent	1<br>Très mécontent
**31**	Êtes-vous content(e) de la qualité de votre propre travail ?	5<br>Très content	4<br>Content	3 Moyennement content	2<br>Mécontent	1<br>Très mécontent

**32**	Êtes-vous content(e) de l'utilité du service que vous rendez aux patientes ?	5<br>Très content	4<br>Content	3 Moyennement content	2<br>Mécontent	1<br>Très mécontent
**33**	Êtes-vous content(e) de la reconnaissance de votre travail par les ***patientes*** ?	5<br>Très content	4<br>Content	3 Moyennement content	2<br>Mécontent	1<br>Très mécontent
**34**	Êtes-vous content(e) de la reconnaissance de la qualité de votre travail par vos ***collègues*** ?	5<br>Très content	4<br>Content	3 Moyennement content	2<br>Mécontent	1<br>Très mécontent
**35**	Êtes-vous content(e) de la reconnaissance de la qualité de votre travail par vos ***supérieurs hiérarchiques*** ?	5<br>Très content	4<br>Content	3 Moyennement content	2<br>Mécontent	1<br>Très mécontent

Questions sur le style de management

**36**	Êtes-vous content(e) de l'application des sanctions ***négatives*** dans votre service ***en général*** ?	5<br>Très content	4<br>Content	3 Moyennement content	2<br>Mécontent	1<br>Très mécontent
**37**	Êtes-vous content(e) de l'application des sanctions ***positives*** dans votre service ***en général*** ?	5<br>Très content	4<br>Content	3 Moyennement content	2<br>Mécontent	1<br>Très mécontent
**38**	Êtes-vous content(e) de la façon dont sont faites les notations ***au niveau de votre service*** pour l'avancement de grade ?	5<br>Très content	4<br>Content	3 Moyennement content	2<br>Mécontent	1<br>Très mécontent
**39**	Êtes-vous content(e) du respect avec lequel vos supérieurs vous traitent ?	5<br>Très content	4<br>Content	3 Moyennement content	2<br>Mécontent	1<br>Très mécontent
**40**	Êtes-vous content(e) des possibilités de participer à la prise de décisions pour résoudre les problèmes d'organisation du travail (est-ce qu'on écoute vos idées, vos propositions quand il y a des problèmes à résoudre dans le travail) ?	5<br>Très content	4<br>Content	3 Moyennement content	2<br>Mécontent	1<br>Très mécontent

**41**	Êtes-vous content(e) des informations qu'on vous donne sur la vie de votre ***service*** (les problèmes, activités, décisions, la gestion financière…) ?	5<br>Très content	4<br>Content	3 Moyennement content	2<br>Mécontent	1<br>Très mécontent
**42**	Êtes-vous content(e) des informations qu'on vous donne sur la vie de votre ***établissement*** (les problèmes, les activités, les décisions, la gestion financière…) ?	5<br>Très content	4<br>Content	3 Moyennement content	2<br>Mécontent	1<br>Très mécontent

43. Classer les 6 dimensions de la satisfaction professionnelle selon l'importance qu'elles ont pour vous :
(Noter 1 pour la plus importante, ainsi de suite, jusqu'à 6 pour la moins importante)

______ : Rémunération /__/

______ : Outils et cadre de travail /__/

______ : Organisation et contenu du travail /__/

______ : Formation et supervision /__/

______ : Satisfaction morale /__/

______ : Style de management /__/

44. Pour résumer, quelle est, dans l'ensemble, votre satisfaction professionnelle?

5<br>Très content	4<br>Content	3 Moyennement content	2<br>Mécontent	1<br>Très mécontent

Attitudes face au changement

45. Pensez-vous que des changements dans la manière de faire le travail seraient une bonne chose pour améliorer les activités de votre service, même si ces changements avaient des conséquences sur votre travail ?
1 Oui 2 Non /__/

46. S'il y a des changements dans la manière de faire le travail, est-ce que vous faites ***spontanément*** des efforts pour vous adapter ?
1 Oui 2 Non /__/

47. S'il y a des changements dans la manière de faire le travail, est-ce que ça vous perturbe ?
1 Oui 2 Non /__/

Existe-t-il d'autres éléments qui n'ont pas été abordés dans ce questionnaire et qui augmentent ou diminuent votre satisfaction professionnelle ?

____

____

____

____

____

____

____

____

____

____

____

____

____

____
