# Supplementary material for: The effects of midwives’ job satisfaction on burnout, intention to quit and turnover: a longitudinal study in Senegal
Source: Hum Resour Health. 2012 Apr 30;10:9. doi: 10.1186/1478-4491-10-9 (PMC3444355; doi:10.1186/1478-4491-10-9)
Supplement: Additional file 3 — Map of the targeted hospital sites (.pdf) (Source of the original map: http://www.afrique-planete.com). [file 1478-4491-10-9-S3.pdf]

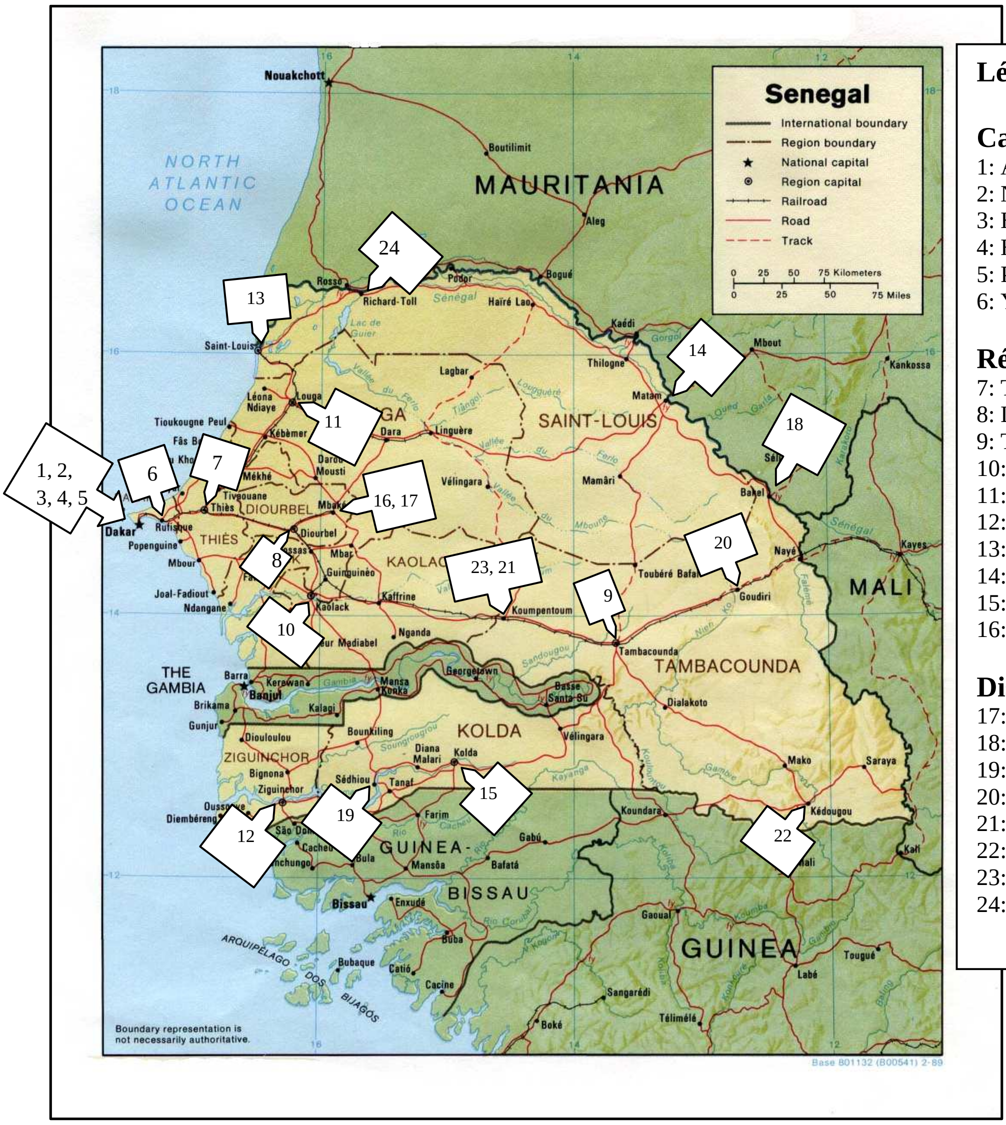

Légende:

Capital:
1: Abass Ndao
2: Nabil Choucaire
3: Hôpital Principal
4: HOGGY
5: Pikine
6: Youssou Mbargane

Régional:
7: Thiès
8: Lubké – Diourbel
9: Tambacounda
10: Kaolak
11: Louga
12: Ziguinchor
13: Saint Louis
14: Ourossogui
15: Kolda
16: Matlaboul Fawzeini

District:
17: Ndamatou
18: Bakel
19: Sedhiou
20: Goudiry
21: Ndioum
22: Kédougou
23: Koungheul
24: Richard Toll
