# Supplementary material for: The effects of midwives’ job satisfaction on burnout, intention to quit and turnover: a longitudinal study in Senegal
Source: Hum Resour Health. 2012 Apr 30;10:9. doi: 10.1186/1478-4491-10-9 (PMC3444355; doi:10.1186/1478-4491-10-9)
Supplement: Additional file 4 — Description of the job satisfaction instrument. [file 1478-4491-10-9-S4.docx]

**Additional file 4**: Description of the job satisfaction instrument

Table of Job Satisfaction Questions and Items

| **Work Facets** | **Items** |
| --- | --- |
| 1Remuneration / Rémunération | q1 : montant du salaire  q3 : primes et indemnités |
| 2 Work environment /  Environnement de travail | q9 : disponibilité du sang pour transfusions  q10 : médicaments pour travail  q11 : consommables pour travail  q12 : protection contre les risques professionnels  q13 : imprimés pour travail |
| 3 Workload /  Charge de travail | q15 : horaires de travail  q16 : charge de travail  q17 : répartition de charge de travail parmi les membres de l’équipe  q20 : répartition du temps de travail entre soins et autres tâches |
| 4 Tasks /  Tâches | q19 : variété des tâches  q21 : adéquation entre tâches et compétences  q22 : niveau de responsabilité professionnelle |
| 5 Working relationships /Entente de travail | q18 : entente entre les membres du service  q34 : reconnaissance de la qualité de votre travail par vos collègues  q35 : reconnaissance de la qualité votre travail par vos superviseurs hiérarchiques  q38 : façon dont sont faites les notations pour l'avancement de grade  q39 : respect avec lequel vos supérieurs vous traitent |
| 6 Continuing education /  Formation continue | q25 : formation que vous continuez à recevoir  q26 : façon de sélection membres service pour des activités de formation |
| 7 Management | q37 : application de sanctions positives dans votre service en général  q40 : possibilités de participer à la prise de décision pour résoudre les problèmes d'organisation du travail  q41 : informations qu'on vous donne sur la vie de votre service  q42 : informations qu'on vous donne sur la vie de votre établissement |
| 8 Moral Satisfaction / Morale | q30 : issue des accouchements et santé des femmes et nouveaux nés  q31 : qualité de votre propre travail |
| 9 Job security /  Stabilité d’emploi | q2 : régularité de versement du salaire  q6 : stabilité d'emploi |
|  |  |

Psychometric qualities of the Job Satisfaction instrument

With the 9-facet model, the fit was found to be acceptable in relation to its complexity and the preliminary nature of the items (Chi square = 681 (341 df) with p<0.0000; CFI = 0.90 and RMSEA = 0.05). Five dimensions had Cronbach alphas slightly below 0.70: remuneration, workload, chores, moral satisfaction and stability. In the case of the last two facets, this is because they are composed of only 2 items. Nevertheless, the 9 factor structure yielded elevated factor score determinacies, ranging from 0.85 to 0.93, thus indicating a good measurement of the factor by the observed indicators.
