# Supplementary material for: The effects of midwives’ job satisfaction on burnout, intention to quit and turnover: a longitudinal study in Senegal
Source: Hum Resour Health. 2012 Apr 30;10:9. doi: 10.1186/1478-4491-10-9 (PMC3444355; doi:10.1186/1478-4491-10-9)
Supplement: Additional file 5 — Complete table of burnout ranges of the study sample. [file 1478-4491-10-9-S5.pdf]

Additional file 5 - Complete table of burnout ranges of the study sample

**Burnout dimensions**	**Range of experienced burnout (Maslach & Jackson, 1996)**	**n**	**%**
EE	Low (≤ 18)	10	5.4
	Average (19-26)	27	14.6
	**High (≥ 27)**	**148**	**80.0**
DP	Low (≤ 5)	39	21.1
	Average (6-9)	39	21.1
	**High (≥ 10)**	**107**	**57.8**
PA*	Low (≥ 40)	96	51.9
	Average (39-34)	66	35.7
	**High (≤ 33)**	**23**	**12.4**

*classified inversely
