# Supplementary material for: The effects of midwives’ job satisfaction on burnout, intention to quit and turnover: a longitudinal study in Senegal
Source: Hum Resour Health. 2012 Apr 30;10:9. doi: 10.1186/1478-4491-10-9 (PMC3444355; doi:10.1186/1478-4491-10-9)
Supplement: Additional file 6 — Complete results from the 2-step linear regressions analyses of job satisfaction scores (independent) and emotional exhaustion scores (dependent). [file 1478-4491-10-9-S6.pdf]

Additional file 6: Results from the 2-step linear regressions analyses of job satisfaction scores (independent) and emotional exhaustion scores (dependent)

STEP 1: Univariate Linear Regressions at $p < 0.10$

Y	X	B Est.	S. E.	t Ratio	p Value
**EE score**	**Job Satisfaction Facet score**				
	1 Remuneration	-3.18	1.12	-2.84	0.01
	2 Work environment	-1.20	1.25	-0.96	0.34
	3 Workload	-4.05	1.67	-2.43	0.02
	4 Tasks	-4.74	1.76	-2.70	0.01
	5 Working relations	-2.40	3.04	-0.79	0.43
	6 Continuing education	-2.04	0.89	-2.30	0.02
	7 Management	-2.49	1.40	-1.78	0.08
	8 Moral satisfaction	-0.99	1.97	-0.50	0.62
	9 Stability	-0.08	2.59	-0.03	0.98

STEP 2: Multivariate Linear Regressions at $p < 0.05$

Y	X	B Est.	S. E.	t Ratio	p Value
**EE score**	**Job Satisfaction Facet score**				
	1 Remuneration	-3.48	1.49	-2.34	0.02
	3 Workload	-1.25	2.06	-0.60	0.55
	4 Tasks	-5.77	2.64	-2.19	0.03
	6 Continuing education	1.33	1.44	0.92	0.36
	7 Management	0.82	1.81	0.46	0.65

Adjusted by: age, tenure, type of institution, educational attainment, rank, employee status, interviewer (T1 : n=185)
