# Supplementary material for: The effects of midwives’ job satisfaction on burnout, intention to quit and turnover: a longitudinal study in Senegal
Source: Hum Resour Health. 2012 Apr 30;10:9. doi: 10.1186/1478-4491-10-9 (PMC3444355; doi:10.1186/1478-4491-10-9)
Supplement: Additional file 7 — Complete results from the 2-step linear regressions analyses of job satisfaction scores (independent) and depersonalisation scores (dependent). [file 1478-4491-10-9-S7.pdf]

Additional file 7: Results from the 2-step linear regressions analyses of job satisfaction scores (independent) and depersonalisation scores (dependent)

STEP 1: Univariate Linear Regressions at $p < 0.10$

**Y**	**X**	**B Est.**	**S. E.**	**t Ratio**	**p Value**
**DP score**	**Job Satisfaction Facet score**				
	1 Remuneration	-1.06	0.70	-1.50	0.13
	2 Work environment	-1.27	0.77	-1.66	0.10
	3 Workload	-2.41	1.03	-2.34	0.02
	4 Tasks	-2.76	1.09	-2.54	0.01
	5 Working relations	-2.15	1.87	-1.15	0.25
	6 Continuing education	-1.13	0.55	-2.05	0.04
	7 Management	-1.47	0.86	-1.72	0.09
	8 Moral satisfaction	-1.64	1.20	-1.36	0.18
	9 Stability	-1.03	1.59	-0.65	0.52

STEP 2: Multivariate Linear Regressions at $p < 0.05$

**Y**	**X**	**B Est.**	**S. E.**	**t Ratio**	**p Value**
**DP score**	**Job Satisfaction Facet score**				
	2 Work environment	-0.56	0.94	-0.60	0.55
	3 Workload	-1.51	1.25	-1.20	0.23
	4 Tasks	-2.08	1.52	-1.37	0.17
	6 Continuing education	-0.08	0.76	-0.11	0.92
	7 Management	0.48	1.21	0.39	0.69

Controlling for: age, tenure, type of institution, educational attainment, rank, employee status, interviewer (T1 : n=185)
