# Supplementary material for: The effects of midwives’ job satisfaction on burnout, intention to quit and turnover: a longitudinal study in Senegal
Source: Hum Resour Health. 2012 Apr 30;10:9. doi: 10.1186/1478-4491-10-9 (PMC3444355; doi:10.1186/1478-4491-10-9)
Supplement: Additional file 8 — Complete results from the 2-step linear regressions analyses of job satisfaction scores (independent) and personal accomplishment score (dependent). [file 1478-4491-10-9-S8.pdf]

Additional file 8: Results from the 2-step linear regressions analyses of job satisfaction scores (independent) and personal accomplishment score (dependent)

	**STEP 1: Univariate analyses at $p < 0.10$**				
**Y**	**X**	**B Est.**	**S. E.**	**t Ratio**	**p Value**
**PA score**	**Job Satisfaction Facet score**				
	1 Remuneration	-0.24	0.57	-0.42	0.67
	2 Work environment	0.82	0.62	1.32	0.19
	3 Workload	0.56	0.85	0.66	0.51
	4 Tasks	1.27	0.89	1.43	0.16
	5 Working relations	1.54	1.52	1.01	0.31
	6 Continuing education	0.38	0.45	0.84	0.40
	7 Management	0.67	0.70	0.95	0.34
	8 Moral satisfaction	1.33	0.98	1.36	0.18
	9 Stability	0.48	1.29	0.37	0.71

	**STEP 2: Multivariate analyses at $p < 0.05$**				
**Y**	**X**	**B Est.**	**S. E.**	**t Ratio**	**p Value**
No facets significant from step 1.					

Controlling for: age, tenure, type of institution, educational attainment, rank, employee status, interviewer (T1 : n=185)
