# Supplementary material for: The effects of midwives’ job satisfaction on burnout, intention to quit and turnover: a longitudinal study in Senegal
Source: Hum Resour Health. 2012 Apr 30;10:9. doi: 10.1186/1478-4491-10-9 (PMC3444355; doi:10.1186/1478-4491-10-9)
Supplement: Additional file 9 — Complete results from the 2-step logistical regressions analyses of job satisfaction (independent) and job search (dependent). [file 1478-4491-10-9-S9.pdf]

Additional file 9: Results from the 2-step logistical regressions analyses of job satisfaction (independent) and job search (dependent)

STEP 1: Univariate analyses at $p < 0.10$

**Y**	**X**	**B Est.**	**S. E.**	**t Ratio**	**p Value**	**OR**	**CI 90%**	
							**Lower**	**Upper**
**Job Search Activities***	**Job Satisfaction Facet****							
	1 Remuneration	-0.93	0.48	3.69	0.06	0.40	0.18	0.88
	2 Work environment	0.00	0.44	0.00	1.00	1.00	0.48	2.07
	3 Workload	0.10	0.44	0.06	0.81	1.11	0.54	2.27
	4 Tasks	0.75	0.48	2.38	0.12	2.11	0.95	4.68
	5 Working relations	-0.41	0.43	0.91	0.34	0.66	0.33	1.34
	6 Continuing education	0.34	0.48	0.50	0.48	1.41	0.64	3.10
	7 Management	0.45	0.47	0.91	0.34	1.57	0.72	3.40
	8 Moral satisfaction	0.17	0.46	0.13	0.72	1.18	0.56	2.50
	9 Stability	-1.88	0.65	8.30	0.00	0.15	0.05	0.45

STEP 2: Multivariate analyses at $p < 0.05$

**Y**	**X**	**B Est.**	**S. E.**	**t Ratio**	**p Value**	**OR**	**CI 95%**	
							**Lower**	**Upper**
**Job Search Activities***	**Job Satisfaction Facet**							
	1 Remuneration	-0.90	0.51	3.07	0.08	0.41	0.15	1.11
	9 Stability	-1.82	0.72	6.37	0.01	0.16	0.04	0.67

Controlling for: age, tenure, type of institution, educational attainment, rank, employee status, interviewer, perception of alternatives (T1 : n=185)
*Hasn't started looking (1) vs Actively job searching (2)   ** Most dissatisfied 25% (1) vs Rest (2)
