# Supplementary material for: The effects of midwives’ job satisfaction on burnout, intention to quit and turnover: a longitudinal study in Senegal
Source: Hum Resour Health. 2012 Apr 30;10:9. doi: 10.1186/1478-4491-10-9 (PMC3444355; doi:10.1186/1478-4491-10-9)
Supplement: Additional file 10 — Complete results from the 2-step logistical regressions analyses of job satisfaction (independent) and turnover (dependent). [file 1478-4491-10-9-S10.pdf]

Additional file 10: Results from the 2-step logistical regressions analyses of job satisfaction (independent) and turnover (dependent)

STEP 1: Uni-variate analyses at $p < 0.10$

**Y**	**X**	**B Est.**	**S. E.**	**t Ratio**	**p Value**	**OR**	**CI 90%**	
							**Lower**	**Upper**
**Turnover***	**Job Satisfaction Facets****							
	1 Remuneration	-0.24	0.61	0.15	0.70	0.79	0.29	2.15
	2 Work environment	-0.89	0.56	2.51	0.11	0.41	0.17	1.04
	3 Workload	-0.25	0.62	0.16	0.69	0.78	0.28	2.18
	4 Tasks	-0.72	0.57	1.63	0.20	0.49	0.19	1.23
	5 Working relations	0.14	0.58	0.06	0.81	1.15	0.45	2.96
	6 Continuing education	-2.24	0.58	14.94	0.00	0.11	0.04	0.28
	7 Management	-1.20	0.59	4.17	0.04	0.30	0.12	0.79
	8 Moral satisfaction	1.21	0.80	2.29	0.13	3.36	0.90	12.51
	9 Stability	17.91	4.32E+03	0.00	1.00	5.97E+07	0.00	.

STEP 2: Multivariate analyses at $p < 0.05$

**Y**	**X**	**B Est.**	**S. E.**	**t Ratio**	**p Value**	**OR**	**CI 95%**	
**Turnover**	**Job Satisfaction Facets**							
	6 Continuing education	-2.13	0.67	10.07	0.00	0.12	0.03	0.44
	7 Management	0.07	0.66	0.01	0.92	1.07	0.29	3.92

Controlling for: age, tenure, type of institution, educational attainment, rank, employee status (T0 : n=226)

*Stay (1) vs Quit (2)   ** Most dissatisfied 25% (1) vs Rest (2)
